# Clinical auditing to improve patient outcomes

**Published:** 2010-12

**Authors:** David Yorston, Richard Wormald

**Affiliations:** Consultant Ophthalmologist, Tennent Institute of Ophthalmology, Gartnavel Hospital, 1053 Great Western Road, Glasgow G12 OYN, Scotland.; Coordinating Editor, Cochrane Eyes and Vision Group (CEVG), International Centre for Eye Health, London School of Hygiene and Tropical Medicine, Keppel Street, London WC1E 7HT.

**Figure:**
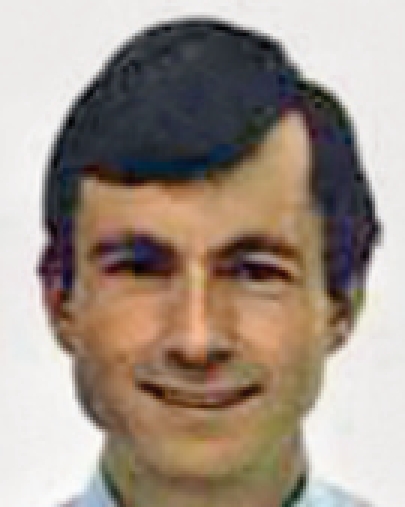


**Figure:**
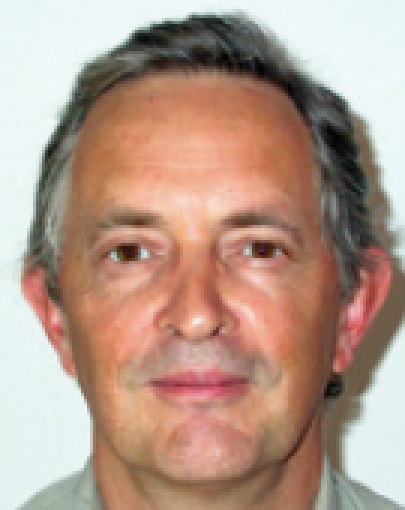


## Auditing: what is it all about?

Clinical audit is about measuring the quality of care we provide against relevant standards. If we are failing to meet these standards, the audit should help us understand the factors that are causing us to fail, so that we can set priorities and make improvements.

Auditing forms part of a cycle of activities:

- Selecting standards (setting our own or adopting existing standards or guidelines).
- Doing the audit (or analysing the results of ongoing monitoring) and identifying where we are failing to meet standards
- Identifying the factors causing us to fail, setting priorities, and taking actions to improve what we do.
- Checking whether we have improved (by doing a full re-audit or by monitoring one or two indicators, for example, visual outcome or patient numbers) and finding other solutions if we have not improved.
- If we have improved, repeating the cycle to identify and address the next set of problems or to measure ourselves against a new set of standards.

Every time an audit cycle is completed, there should be further improvement in patient care.

Audit and research are different, although there can be overlap. Audit cannot be used to show that one technique or treatment is better than another - this usually requires prospective randomised controlled trials. If differences in outcome are observed, these may be the result of many different factors: case selection, natural history, and other factors such as resources or training. These may be precisely those factors that your audit is trying to understand.

**Figure:**
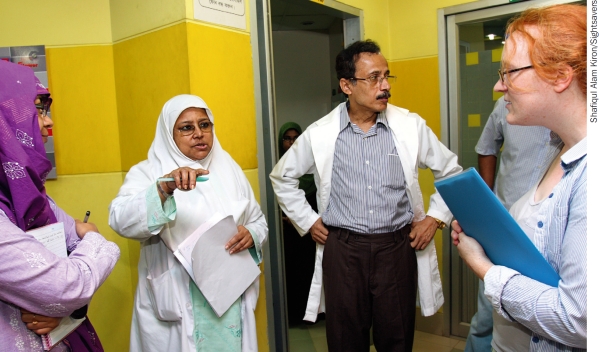
For auditing to have an impact on patient care, it is important to share the results with all staff members on a regular basis. BANGLADESH

Shafiqul Alam Kiron/Sightsavers

## What could I gain from doing an audit?

Here are a few examples:

**Evidence for resources.** Audit is a very powerful tool for providing evidence for the need for specific resources to increase quality standards or performance. This could be another member of staff to reduce waiting times in a clinic or a specific piece of equipment (for instance an A scan for biometry to improve refractive outcomes after cataract surgery).

**Feedback for the community outreach team.** Auditing and monitoring of patient details can identify trends in attendance for eye surgery. For example, attendance rates for referrals may differ according to community members' age, gender, visual status, or area of residence. Once understood, these can be addressed by the community outreach team.

**Understanding the impact of changes at the hospital.** If done over a sufficiently long period, auditing and monitoring of patient attendance, in particular, will reflect the effects of any changes in the quality of counselling or care provided, or changes in how the hospital is run (such as routine postoperative counselling or cleaner waiting rooms).

**‘Every time an audit cycle is completed, there should be further improvement in patient care’**

**Identification of high-risk groups.** Auditing and monitoring the preoperative health assessment and visual acuity of patients and comparing these against the surgical and visual outcome after surgery can identify patients who are at higher risk of complications. If these are predicted, the operating theatre can be prepared for them, and the ophthalmologist can ensure that operations are carried out by an experienced surgeon who is able to deal with any complication that occurs.

**Measuring quality of care.** Some treatment outcomes are very long term. For example, it is difficult to audit the results of treatment for open-angle glaucoma, as it can take years to know the final outcome. However, you can audit the quality of your care against standards, whether these are standards set by others (e.g. national or international bodies) or those which you set yourself. For example, what is the complication rate after trabeculectomy (and how does this compare with other hospitals in your country)?

## How do I get started?

- You need to decide what question you want to answer. What is the issue, problem, or question of concern? These can be driven by national or local quality requirements, such as those set by a funding agency, but may also be specific to a problem you wish to deal with. Complaints or critical incidents (events or circumstances that caused or could have caused unplanned harm, suffering, loss, or damage) can be a useful way of identifying problems that need to be addressed.
- Create a dummy report of your intended results. Say you want to audit the outcomes of corneal ulcer treatment. Your final report might include things like treatment given, average time to healing, average length of admission, culture and gram stain results, and final vision. You decide what you want in the final report and this will determine what data you need to collect in order to generate the desired report. This means you must have a plan for the analysis of the data you collect; it also ensures you collect it in a format that is as easy as possible for you to analyse.
- Discuss the audit with all your staff, particularly those who will have to collect data, so that they are informed and understand the reason for the audit. All members of the eye care team involved in the collecting of data need to be motivated to collect the data consistently. It is vital that they feel there is some purpose to it and that ultimately they and their patients will benefit.
- Develop the form you will use to collect the data. The aim should be to collect **sufficient data** (name, address, age, sex, date of surgery/admission, initial and final visual acuity, treatment/ surgery details, and complications) on **all patients** and to do this for a **long time** so trends can be identified and progress monitored.
- Pilot the form on a number of patients, or for a given time. Review the results of the pilot. is there information you do not need, or is there missing data? Can the staff collect the data without problems?
- Based on the pilot, revise the data collection form. An important principle here is to be sure you **only collect data which you intend to analyse.** There is a tension between monitoring for management purposes, which requires minimum data on all patients over a long time, and the needs of eye surgeons to have detailed feedback for clinical purposes to help them refine their surgical and diagnostic skills - and for which they need a lot more data on fewer patients. One solution is for the surgical team to plan additional data collection projects to give them the detailed feedback they need, for example having a detailed monitoring form for every fifth or tenth patient.
- Start the audit and data collection.
- Analyse the data at predefined intervals; you would normally ‘freeze’ or ‘lock’ the database before each analysis.
- Provide regular feedback to all those involved. Hold regular meetings where you can give feedback on the findings and ensure that all staff are invited to attend. At the very least, there should be representatives from nursing, administration, finance, community outreach, pharmacy and supplies, as well as the surgeons and ophthalmologists. If poor outcomes are the result of poor case selection, then the community outreach team need to know. If poor outcomes are caused by endophthalmitis, and the surgeons decide to use intracameral cefuroxime as a prophylaxis, then the pharmacists need to know so that they can ensure the drug is available and that it is made up in the correct dilution.
- Use the audit to inform future policy and decision making so as to improve the eye care service.

## Suggestions for successful auditing

- Minimise the extra work required. If possible, the information required for auditing should be integrated with the routine recording of clinical data. This can be done by using a standard form. This ensures that the necessary details are recorded and makes it simple for a clerical worker to transfer them to a computer. The form is placed in the patient's file and becomes the clinical record of the operation and postoperative care.
- Data should be collected on all patients, even those in whom a good outcome is impossible owing to pre-existing co-morbidity, e.g., previous glaucoma surgery. Although this means that a higher proportion of eyes will have a poor outcome, it permits a more reliable estimate of trends within the clinic.
- An audit programme should also monitor safety and include mechanisms for identifying common errors or mistakes as well as rare and more serious adverse outcomes. Monitoring complaints and critical incidents (for example, if a patient's life was in danger) are two important means of doing this. Audit for these types of outcomes should be routine and integrated into everyday activity.

In conclusion, the aim of an audit is not to identify a guilty person and then punish him or her. We know that we all make mistakes and we all have complications. The reason for auditing is to identify the problems, to learn from them, and to try to avoid making the same mistakes again and again.

**Case study: an example of auditing that worked**

The Kikuyu Eye Unit team audited all cataract operations over a twelvemonth period. They were able to identify that patients who experienced vitreous loss had worse outcomes. They also showed that patients who were blind preoperatively (<3/60 in both eyes) did worse than those who had a unilateral cataract and still retained useful vision in the other eye. They found that most of the patients who had a poor final vision had coexisting eye problems, such as glaucoma or corneal scar.

Armed with these results, all the surgeons in the team were retrained to manage vitreous loss more safely. Patients with bilateral blinding cataract were allocated to a senior surgeon. Perhaps most importantly, there was a cultural change: the team was no longer concerned solely with the quantity of operations, but also with the outcome. As a result, they became more selective and operated on fewer patients who had other blinding conditions and were highly unlikely to benefit from surgery.

The auditing results were shared with staff at regular intervals throughout the year. During this time, there was a highly significant trend showing a steady increase in the number of good outcomes and a decrease in the number of poor outcomes. Without the audit, the eye surgeons would not have known what needed to be changed and the improvement would never have happened.
